# Supplementary material for: Neuro-Axonal Damage and Alteration of Blood–Brain Barrier Integrity in COVID-19 Patients
Source: Cells. 2022 Aug 10;11(16):2480. doi: 10.3390/cells11162480 (PMC9406414; doi:10.3390/cells11162480)
Supplement: Supplementary file 1 [file cells-11-02480-s001.zip › cells-1829306-supplementary.pdf]

Table S1. Plasma NfL levels on hospital admission, comorbidities and maximal ventilation needed during hospitalization in COVID-19 patients.

Patient	Comorbidity	Ventilation	Plasma NfL (pg/mL)	CSF NfL (pg/mL)	CSF MMP-2 (OD/mm²)	CSF MMP-9 (OD/mm²)
**1**	none	VMK	29.9	330	50.4	11.0
**2**	diabetes	NIV	83.9	889	96.0	19.1
**3**	arterial hypertension, cancer	AA	27.2	622	87.1	19.9
**4**	none	VMK	-	1591	79.0	15.2
**5**	arterial hypertension, diabetes	NIV	-	7961	126.3	11.5
**6**	chronic pulmonary disease	AA	19.8	318	-	-
**7**	cancer	IOT	90.3	4998	80.3	114.4
**8**	none	NIV	103.0	18,555	92.4	21.9
**9**	arterial hypertension, chronic pulmonary disease, renal	VMK	-	6661	84.3	10.2
**10**	arterial hypertension, diabetes	NIV	59.5	8497	84.5	67.1
**11**	arterial hypertension, diabetes	IOT	96.7	1720	88.5	15.7
**12**	cancer	VMK	-	266	72.4	15.6
**13**	none	AA	14.4			
**14**	none	NIV	21.8			
**15**	arterial hypertension	NIV	14.2			
**16**	cancer	VMK	14.4			
**17**	none	AA	11.2			
**18**	none	IOT	28.6			
**19**	arterial hypertension	AA	34.0			
**20**	none	NIV	27.1			
**21**	arterial hypertension	NIV	41.4			
**22**	arterial hypertension, cardiovascular	NIV	22.3			
**23**	none	AA	27.6			
**24**	cardiovascular, cancer	IOT	123			
**25**	chronic pulmonary disease	NIV	29.4			
**26**	none	VMK	26.7			
**27**	none	VMK	8.88			
**28**	none	VMK	20.9			
**29**	none	IOT	39.0			
**30**	none	AA	13.9			
**31**	arterial hypertension, cardiovascular	AA	29.0			
**32**	none	IOT	42.6			
**33**	cancer	NIV	76.4			
**34**	arterial hypertension	NIV	10.3			
**35**	none	AA	10.2			
**36**	arterial hypertension	NIV	10.9			
**37**	arterial hypertension	AA	17.8			
**38**	none	AA	4.43			
**39**	none	IOT	16.8			
**40**	arterial hypertension	NIV	86.3			
**41**	arterial hypertension, cancer	NIV	42.2			
**42**	arterial hypertension	AA	4.96			
**43**	arterial hypertension	NIV	59.0			
**44**	cardiovascular, diabetes	IOT	31.1			
**45**	none	VMK	32.5			
**46**	diabetes	NIV	11.7			

**47**	none	NIV	14.9
**48**	arterial hypertension	IOT	15.3
**49**	arterial hypertension, chronic pulmonary disease	NIV	28.1
**50**	none	AA	0.221
**51**	none	NIV	36.4
**52**	none	AA	19.9
**53**	none	AA	15.1
**54**	none	AA	4.56
**55**	none	VMK	39.3

NfL: neurofilament light chain, CSF: cerebrospinal fluid, MMP-2: matrix metalloprotease-2, MMP-9: matrix metalloprotease-9, OD: optical density, AA: ambient air, VMK: Venturi oxygen mask, NIV: noninvasive ventilation, IOT: invasive mechanical ventilation through orotracheal intubation.

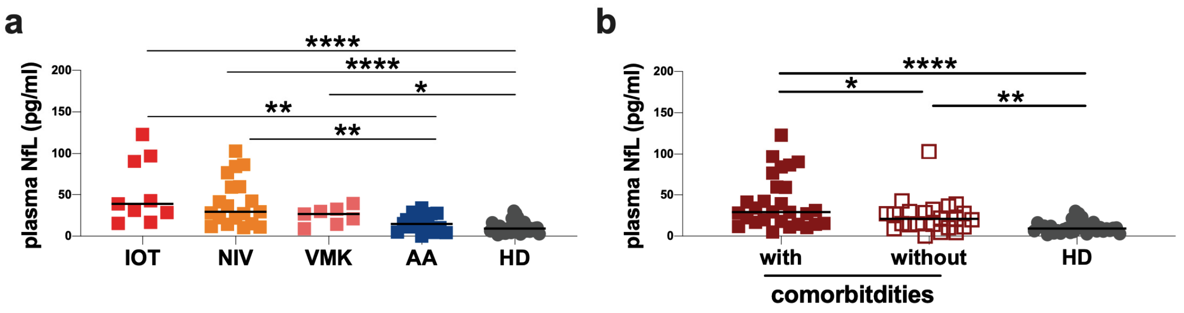

Figure S1. Evaluation of plasma NfL levels on hospital admission in COVID-19 patients stratified according to the presence of comorbidities and maximal ventilation needed during hospitalization. NfL: neurofilament light chain, AA: ambient air, VMK: Venturi oxygen mask, NIV: noninvasive ventilation, IOT: invasive mechanical ventilation through orotracheal intubation. ****: $p < 0.0001$; **: $0.001 < p < 0.01$; *: $0.01 < p < 0.05$.
